# Supplementary figures and images for: Exploring the application of AI in the education of children with autism: a public health perspective
Source: Front Psychiatry. 2025 Jan 28;15:1521926. doi: 10.3389/fpsyt.2024.1521926 (PMC11811491; doi:10.3389/fpsyt.2024.1521926)

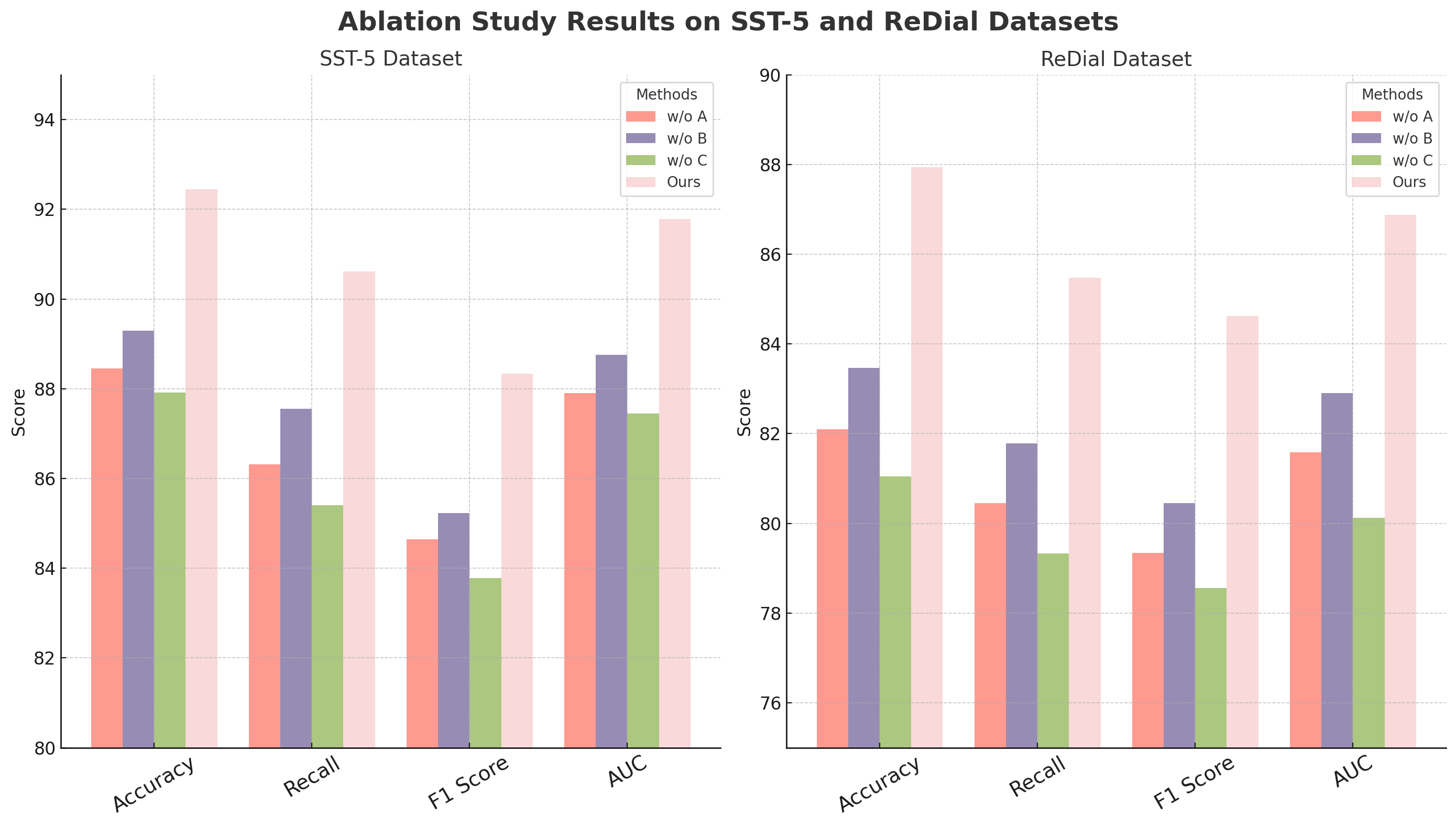

Supplement: Supplementary file 1 [file Image1.png]

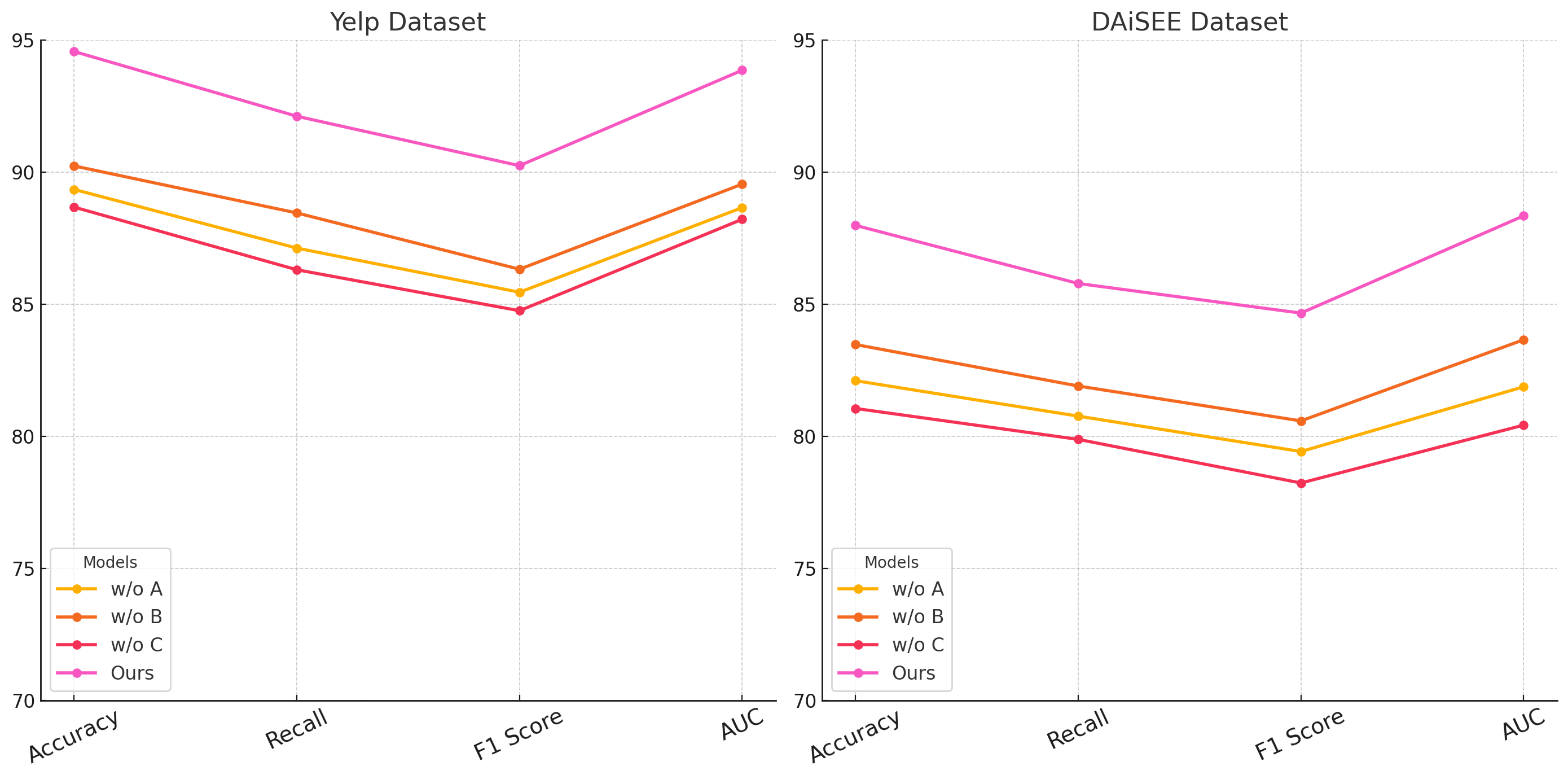

Supplement: Supplementary file 2 [file Image2.png]

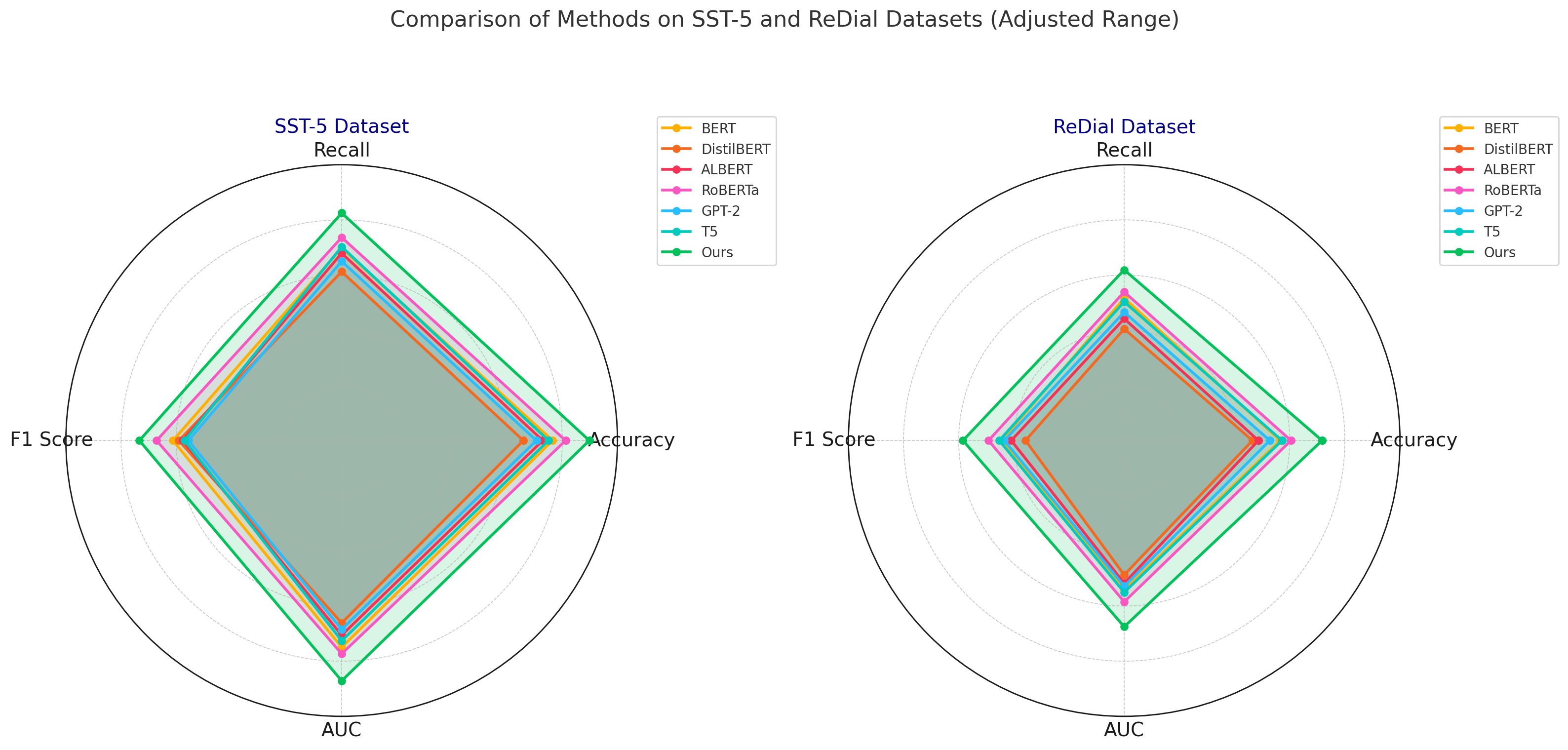

Supplement: Supplementary file 3 [file Image3.png]

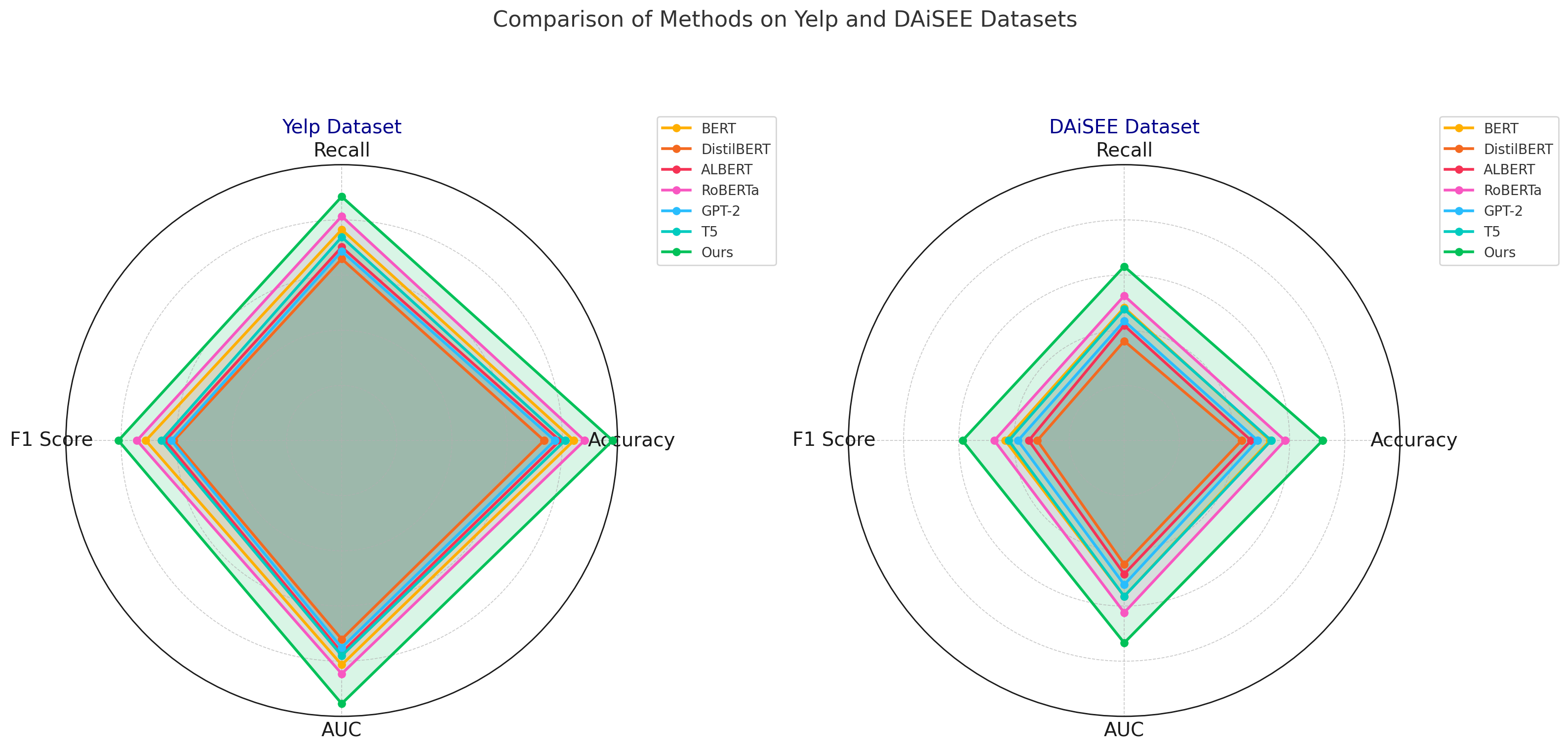

Supplement: Supplementary file 4 [file Image4.png]

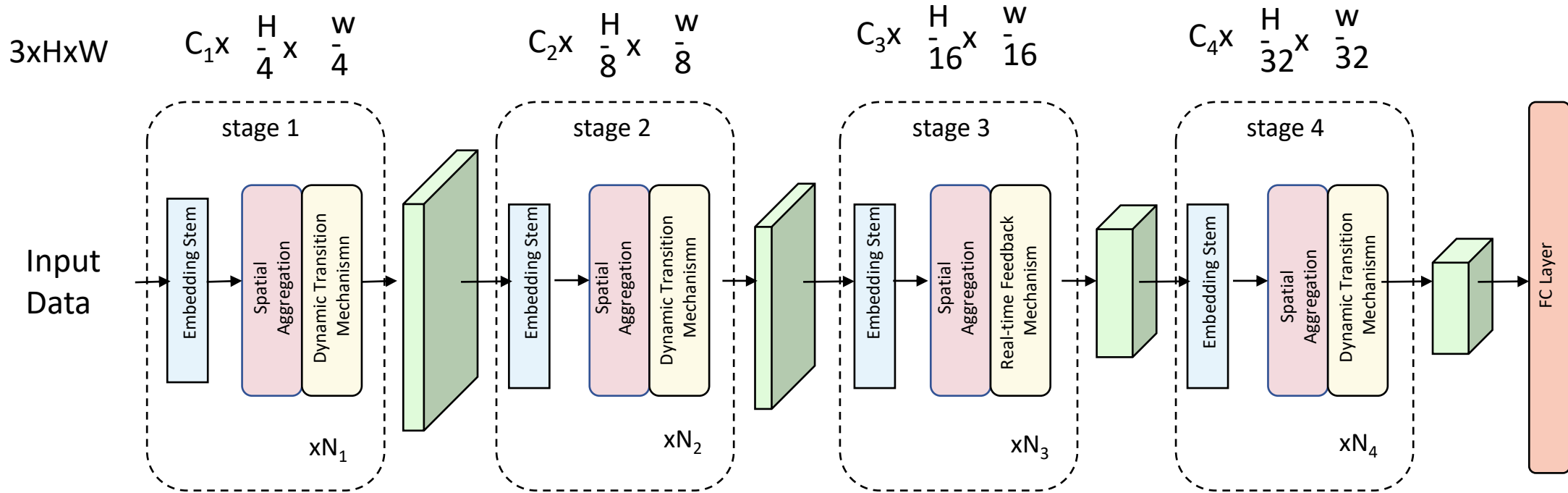

Supplement: Supplementary file 5 [file DataSheet1.pdf]

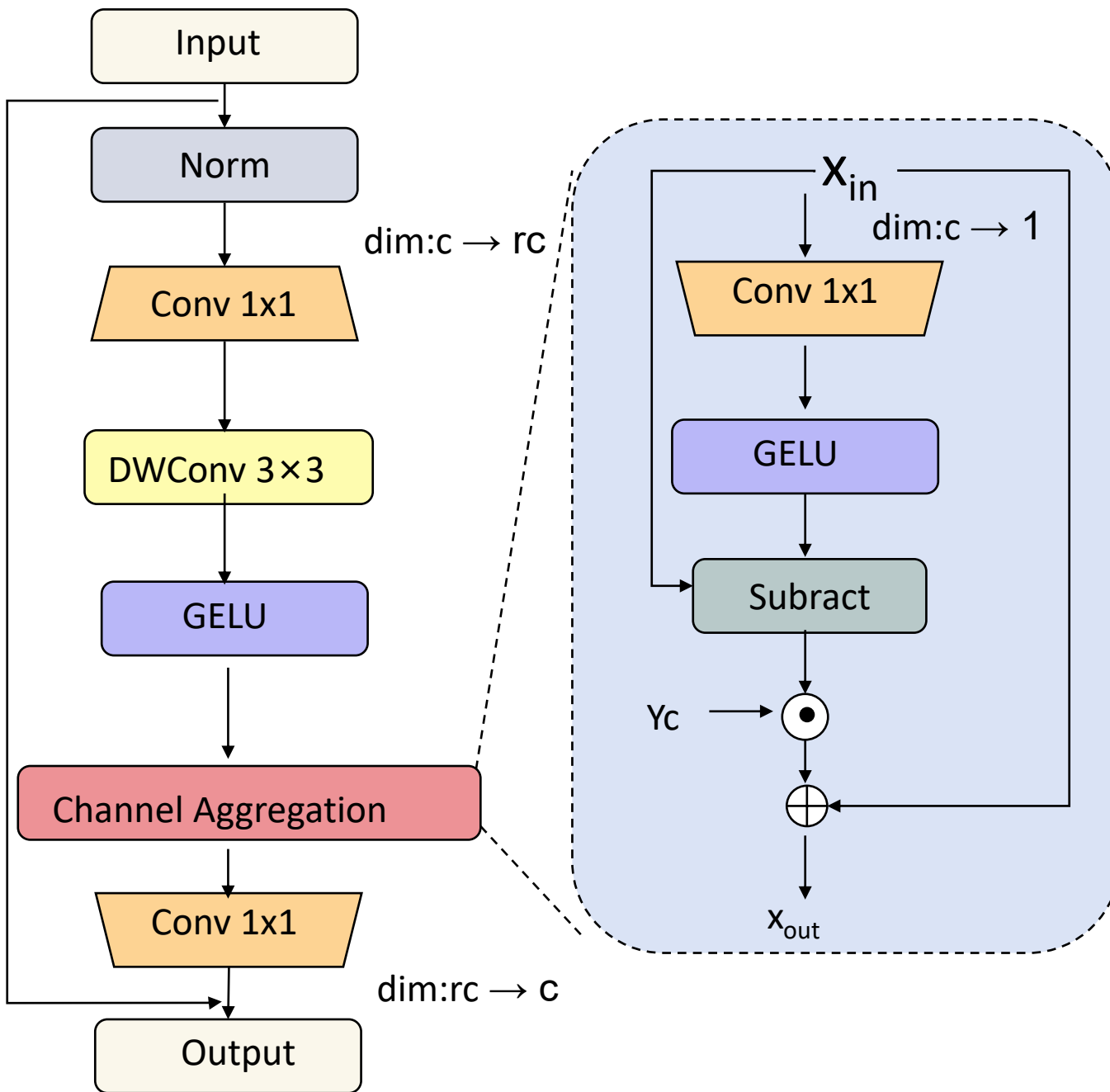

Dynamic Transition Mechanism

Supplement: Supplementary file 7 [file DataSheet3.pdf]
