# Supplementary material for: Exploring the application of AI in the education of children with autism: a public health perspective
Source: Front Psychiatry. 2025 Jan 28;15:1521926. doi: 10.3389/fpsyt.2024.1521926 (PMC11811491; doi:10.3389/fpsyt.2024.1521926)
Supplement: Supplementary file 6 [file DataSheet2.pdf]

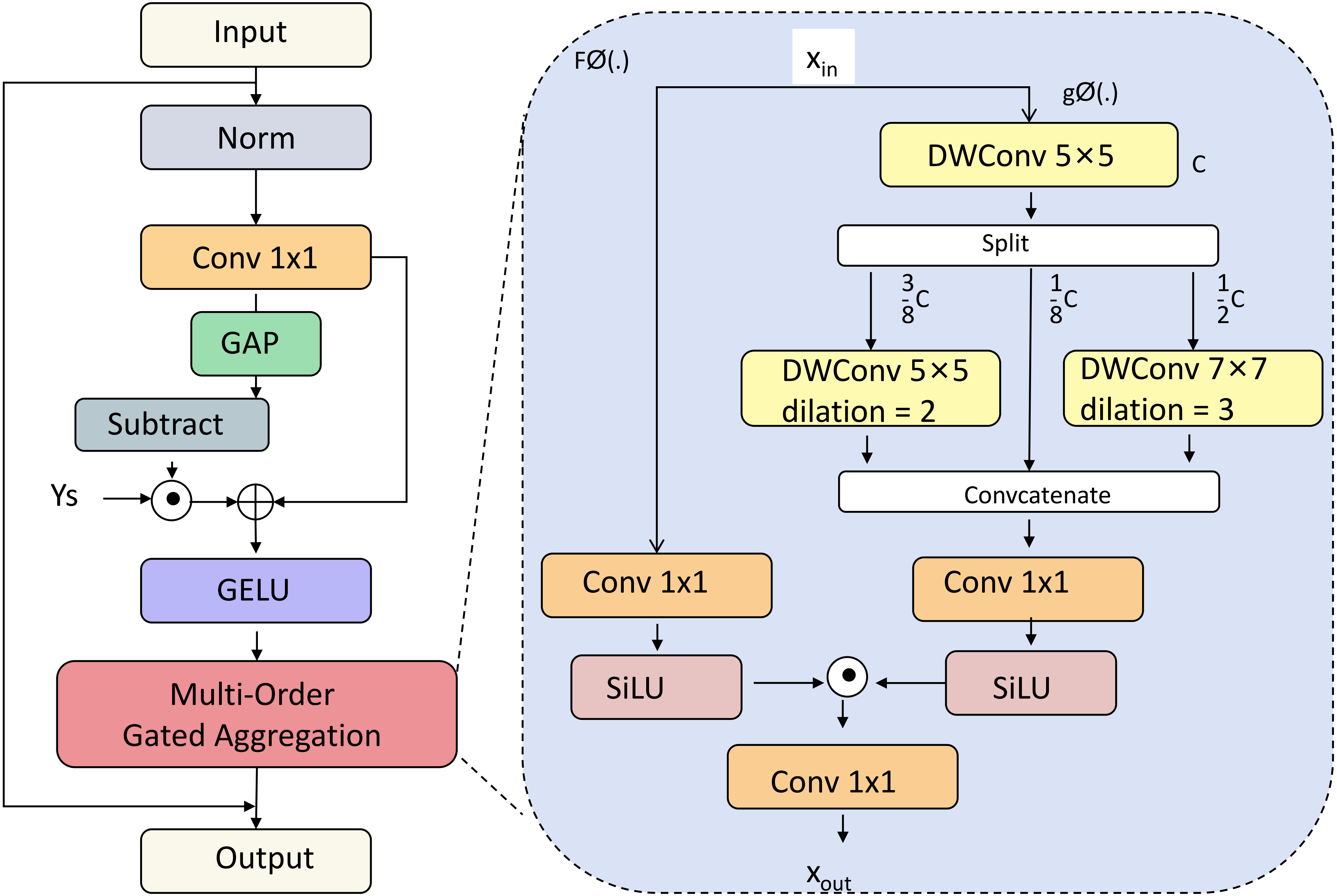

Latent Interaction State Representation
